# Choroid Plexus Acts as Gatekeeper for TREM2, Abnormal Accumulation of ApoE, and Fibrillary Tau in Alzheimer’s Disease and in Down Syndrome Dementia

**DOI:** 10.3233/JAD-181179

**Published:** 2019-05-07

**Authors:** Ruma Raha-Chowdhury, James W. Henderson, Animesh Alexander Raha, Romina Vuono, Anastasia Bickerton, Elizabeth Jones, Robert Fincham, Kieren Allinson, Anthony Holland, Shahid H. Zaman

**Affiliations:** a Cambridge Intellectual and Developmental Disabilities Research Group, Department of Psychiatry, Cambridge, UK; b John van Geest Centre for Brain Repair, Department of Clinical Neuroscience, University of Cambridge, Cambridge, UK; c Clinical Pathology, Addenbrookes Hospital, Cambridge University Hospitals NHS Foundation Trust, Cambridge, UK; d Cambridgeshire and Peterborough Foundation NHS Trust, Cambridge, UK

**Keywords:** Alzheimer’s disease, blood-brain barrier, blood-CSF barrier, choroid plexus, Down syndrome, high-risk haplotype, neuroinflammation, tau trafficking, TREM2, white matter tract

## Abstract

**Background:**

Genetic factors that influence Alzheimer’s disease (AD) risk include mutations in *TREM2* and allelic variants of *Apolipoprotein E*, influencing AD pathology in the general population and in Down syndrome (DS). Evidence shows that dysfunction of the choroid plexus may compromise the blood-cerebrospinal fluid (CSF) barrier, altering secretary, transport and immune function that can affect AD pathology.

**Objective:**

To investigate the genotype and phenotype of DS individuals in relation to choroid plexus damage and blood-CSF barrier leakage to identify markers that could facilitate early diagnosis of AD in DS.

**Methods:**

To assess allele frequency and haplotype associations *ApoE, Tau, TREM2*, and *HLA-DR* were analyzed by SNP analysis in DS participants (*n* = 47) and controls (*n* = 50). The corresponding plasma protein levels were measured by ELISA. Postmortem brains from DS, AD, and age-matched controls were analyzed by immunohistochemistry.

**Results:**

Haplotype analysis showed that individuals with *Tau H1/H1* and *ApoE* *ɛ4* genotypes were more prevalent among DS participants with an earlier diagnosis of dementia (17%) compared to *H1/H2* haplotypes (6%). Plasma TREM2 levels decreased whereas phospho-tau levels increased with age in DS. In AD and DS brain, insoluble tau and ApoE were found to accumulate in the choroid plexus.

**Conclusion:**

Accumulation of tau and ApoE in the choroid plexus may increase the oligomerization rate of Aβ_42_ and impair tau trafficking, leading to AD pathology. We have identified a high-risk haplotype: *ApoE* *ɛ4, Tau/H1*, and *TREM2/T,* that manifests age-related changes potentially opening a window for treatment many years prior to the manifestation of the AD dementia.

## INTRODUCTION

Down syndrome (DS) is the most common human aneuploidy associated with intellectual disability and early neurodegeneration. It is due to triplication of all or part of chromosome 21, where genes, including the amyloid precursor protein (*APP*) gene, that play key roles in the pathogenesis of Alzheimer’s disease (AD) are encoded. People with DS have an age-dependent increased risk for developing dementia; effectively all adults with DS over 40 years of age have excessive numbers of senile plaques (SPs), consisting primarily of amyloid-β peptide (Aβ) [1, 2]. The overexpression of the *APP* gene may lead to excess Aβ deposition, occurring decades earlier in people with DS compared to those in the typically developing population with AD [3]. In addition to the appearance of SPs and neurofibrillary tangles (NFTs) surrounded by Aβ plaques, aggregation of neuritic threads (NTs) and neuropils (NPs) have also been found in DS brain [4, 5]. The microtubule associated protein tau (MAPT) becomes hyperphosphorylated and aggregated, leading to the formation of NFT. Although dysfunction of amyloid-β protein precursor (AβPP) processing is believed to be the key upstream factor in the pathogenesis of AD [6], neuroinflammation and activation of innate immunity are considered early events in the genesis of AD and in DS dementia. In DS and in late-onset AD, neuroinflammation has been linked to both the exacerbation of SP and NFT pathology, as well as the clearance of Aβ from Aβ plaques [7–9]. The main cell types involved in neuroinflammatory responses in the brain are microglia and to a lesser extent astrocytes [10, 11]. Microglia in the vicinity of the pathological hallmarks of AD become activated and release a variety of inflammatory cytokines and chemokines [12]. Genome-wide association studies have shown that a rare mutation of Triggering Receptor Expressed on Myeloid cells (*TREM2-R47H* mutation) correlates with a heightened risk of developing AD [13–16]. *In vitro* studies indicate that TREM2 deficiency reduces the efficacy of Aβ clearance mechanisms and thus can contribute to AD pathogenesis [17]. We have recently provided evidence that in soluble TREM2 (sTREM2), the cleaved ectodomain of TREM2, protein levels decline with age and disease progression in the brain and in the sera of people with DS [18].

Another key protein involved in AD and in the risk of AD in DS is Apolipoprotein E (*ApoE*). This gene is a major genetic risk factor for AD; 60–80% affected individuals have at least one *ApoE* *ɛ4* allele [19, 20]. *ApoE* *ɛ4* carriers develop Aβ plaques earlier than non-carriers. ApoE has been shown to impair the metabolism of Aβ and consequently decrease the rate of Aβ clearance from the brain [21–24]. Apolipoproteins, including ApoE and ApoJ, bind to TREM2 and the uptake of Aβ-lipoprotein complexes was reduced in macrophages carrying the *TREM2 /R47H* mutation [25]. However, the molecular mechanism and cellular pathways by which TREM2, tau, and ApoE influences Aβ clearance and plaques deposition in AD and in DS remain poorly understood.

We and others have previously provided evidence that dysfunction of the choroid plexus (CP), which results in altered secretory, transport, immune, and barrier functions, contributes to normal aging and the age-associated AD [26, 27]. The CP contains fenestrated capillaries surrounded by tightly connected choroid plexus epithelial (CPE) cells that form the blood-cerebrospinal fluid barrier (BCSFB). The major tasks of the CPE cells include cerebrospinal fluid (CSF) production, removal of toxic waste products, and acting as gatekeepers of the brain by ensuring the presence of resident inflammatory cells [28]. The tight junctions located between the apical parts of the CPE cells form part of the BCSFB, which is crucial for the homeostatic regulation of the brain microenvironment along with the blood-brain barrier. Morphological changes, such as atrophy of the epithelial cells and thickening of the basement membrane, suggest altered CSF production occurs with aging and in AD. Additionally, this structure is important as an inflammatory sensor that detects signals originating from both the peripheral and the central nervous system [29, 30].

This is a longitudinal study; DS participants (*n* = 47) were followed for six years by the Cambridge Developmental Disabilities Research Group, Cambridge, UK [31]. The aim of the study was to evaluate both genotype and phenotype (plasma protein expression by ELISA), and to compare these findings with positron emission tomography (PET) imaging of DS participants as previously reported [32]. Our second aim was to identify disease specific haplotypes and finally to compare the AD pathology involved in DS brain (in postmortem brain samples) with age matched AD and control brain. All DS participants had clinical evaluations and MRI and PET scanning to the aid the diagnosis of dementia [31, 32].

Our new findings, supported by ongoing PET/MRI neuroimaging data, could be developed into potential biomarkers that could identify high-risk individuals suitable for pre-emptive therapy many years prior to the manifestation of the dementia phenotype.

## MATERIALS AND METHODS

### Ethics and participants

Ethics and research and development (R&D) approvals were granted by the National Research Ethics Committee of the East of England – Norfolk and Cambridgeshire and Peterborough NHS Foundation Trust, respectively, (Project ref no: REC:15/WM/0379). Written consents were obtained from controls (*n* = 50) and all adults with DS participants (*n* = 47) with capacity to consent. Verbal assent was obtained from participants with DS lacking capacity to provide written assent, which was provided instead by an appointed consultee, in accordance with the Mental Capacity Act (2005) of the UK.

### Assessment of dementia status

This was undertaken as described previously using the CAMDEX-DS informant interview and the CAMCOG-DS neuropsychological assessment [31].

### Brain tissues

Human postmortem brain tissues from controls (mean age 60±15 years), DS (mean age 50±15 years) and AD (mean age 82.0±8.0 years) (N = 10 in each group) were provided by the Cambridge Brain Bank (Supplementary Table 1). Cambridge Health Authorities Joint Ethics Committee granted ethical approval for use of human brain tissue and serum samples (Project ref no: REC:15/WM/0379).

Ethical approval for all procedures performed in studies involving human participants were in accordance with the ethical standards of the institutional and/or national research committee and with the 1964 Helsinki declaration and its later amendments or comparable ethical standards.

### Blood and serum/plasma collection

Whole blood, serum, and plasma samples from human controls (*n* = 50) and DS individuals (*n* = 47) were collected for DNA and protein analysis at our Research Centre during a six-year period from July 2012 to June 2018. Blood samples were collected into EDTA tubes for DNA and plasma and serum blood collection tubes. Serum and plasma were separated immediately by centrifugation at 2465 g for 6 min at 4°C, aliquoted, and stored at –80°C until analysis. Biochemical and hematological profiles were analyzed by pathology laboratories at Addenbrooke’s Hospital, Cambridge University NHS Foundation Trust, Cambridge.

### Single nucleotide polymorphism analysis and genotyping

Single nucleotide polymorphism (SNP) genotyping for *TREM2* SNP (rs75932628, *C-T* encoding the *R47H* variant), *Tau* (rs 9468, tagging *MAPT, H1* versus *H2* haplotypes) and *HLA-DR* (rs3129882 *A/G* polymorphism) were performed using the TaqMan® allelic discrimination assay on an HT7900 sequence detection system (Applied Biosystems), according to the manufacturer instructions. Genotyping success rates were >96%. There were no inconsistencies amongst 92 samples genotyped in duplicate. *APOE* (*ɛ*2, *ɛ*3, and *ɛ*4) genotypes were analyzed by the Genotype Facilities, East Anglia Medical Genetics Service, Addenbrooke’s Hospital, Cambridge University Hospitals NHS Foundation Trust, Cambridge.

### Antibodies

The following primary antibodies were used: mouse monoclonal (mAb) anti-TREM2 (ab 201621, MM0942-42E14) and rabbit mAb anti-TREM2 (ab209814) and other antibodies (Table S2) from Abcam (Cambridge, UK). The mAb anti-Aβ antibody (6E10) (Signet Laboratory) has been described previously [18]. Other antibodies used in this study including rabbit mAb anti-Aβ antibodies (Abcam ab201060) can be found in Supplementary Table 2. The following secondary antibodies were used: i.e., biotinylated goat anti-rabbit-Ig and biotinylated horse anti-mouse (both from Vector Laboratories, 1:250 for IHC); Alexa Fluor 568-labelled donkey anti-mouse-Ig, Alexa Fluor 488-labelled donkey anti-rabbit-Ig; and Alexa Fluor 568-labelled donkey anti-goat-Ig (all from Invitrogen, 1:1000 for immunofluorescence).

### Solid phase enzyme linked immunosorbent assay (ELISA)

To quantify the concentrations of the soluble extracellular domain of Triggering Receptor Expressed on Myeloid cells 2 (sTREM2) in human EDTA-plasma samples, we adapted an anti-TREM2 ELISA system similar to that reported by Kleinberger et al. [33]. For the detection of sTREM2, plates were incubated overnight with mAb anti-TREM2 capture antibody (1 μg/ml) (Abcam, Cambridge). The following day the plates were washed three times with washing buffer (0.05% Tween 20 in 0.1 M phosphate buffer saline (PBS) pH 7.4) and blocked in blocking solution (1% BSA and 0.05% Tween 20 in 0.1M PBS) for 2 hours (2h) at room temperature (RT). After blocking, the plates were washed three times with washing buffer and loaded with 10 μl plasma into 90 μl blocking solution and incubated 4 h at RT. A recombinant human TREM2 protein (Life Technologies, Grand Isle, NY) was diluted in assay buffer in a twofold serial dilution and used for the standard curve with a concentration range of 1000, 500, 250, 125, 62, 31, 15, and 0 pg/ml. After 4 h of incubation, the samples were removed and the plates were washed three times for 5 min with washing buffer before incubation for 2 h at room temperature for detection with a biotinylated rabbit monoclonal anti-human TREM2 antibody (1 μg/ml) diluted in blocking buffer. After three further washing steps, the plates were incubated with anti-rabbit HRP-conjugated secondary antibody (1:4000) for 1 h followed by three washes. 100 μl of 1-Step ULTRA tetramethylbenzidine (TMB-ELISA, ThermoScientific) was added for ∼30 minutes at room temperature. Finally, 100 μl of 2 M H_2_SO_4_ was added to quench the reaction. The plates were read with an Infinite m200 plate reader (Tecan) at 450 nm.

Standard curve linearity and inter-plate and inter-day variability for the sTREM2 ELISA were determined using dedicated plasma sample anchors for all plates. The specificity of the ELISA system used was further validated by anti-TREM2 immunoblotting showing a high degree of correlation between the ELISA readings and immunoreactivity on the immunoblot using these antibodies and an independent anti-TREM2 antibody, mouse anti-TREM2 2B5 (R&D Systems, Minneapolis, MN). The plasma was stored at –70°C until used for further assays. Similar methods were followed for amyloid-β_42_ human ELISA kit (Catalog number: KHB1 3544, Thermo Fisher scientific), Aβ_40_ (Catalog number: KHB1 3482, ThermoFisher scientific), and ApoE (Abcam, Ab108813) with the ApoE kit recognizing ApoE *ɛ*2, ApoE *ɛ*3, and ApoE *ɛ*4 isoforms.

### Measurement of total-tau and phospho-tau by ultrasensitive immunoassay technique

Plasma total-Tau (t-Tau) was measured with the Human Total Tau kit (Simoa™ Tau 2.0 Kit, Quanterix, Lexington, MA) in accordance with an updated version of the previously described assay that uses a monoclonal capture antibody that reacts with a linear epitope in the mid region of all tau isoforms (phosphorylated and nonphosphorylated) and a detection antibody that reacts with a linear epitope in the N-terminus of t-Tau or phospho-tau (p-Tau). All samples were analyzed in triplicate on one occasion. (Intra-assay co-efficients of variance of the measurements were less than 5%.) All cases and controls were evenly distributed on the plate.

We measured levels of p-Tau in plasma by modifying an ultrasensitive immunoassay technique to quantify plasma p-Tau phosphorylated at threonine 181 (p-tau181) instead of human total tau (Simoa™ Tau 2.0 Kit, Quanterix, Lexington, MA). We have used a monoclonal capture antibody that reacts with a linear epitope in the mid region of all tau isoforms and a detection antibody that reacts with an epitope in the N-terminal region of t-Tau. Instead of this detection antibody against t-Tau, we employed anti-human PHF-tau monoclonal antibody AT270 (Thermo Fisher Scientific, Rockford, IL, USA) as the detection antibody for p-Tau181 immunoassay. This set of capture and detection antibodies specifically reacts with p-Tau181 without reacting with other phosphorylated tau variants. All plasma samples were diluted 4 times with the Tau 2.0 sample diluent prior to the assays, to minimize matrix effects. To eliminate inter-assay variability as a confounding factor, all plasma samples belonging to the same cohort were run in duplicate on the same day with the same set of standards. The relative concentration estimates of plasma p-Tau181 were calculated according to a standard curve.

### Immunofluorescence

Brain sections were blocked using blocking buffer (0.1 M PBS, 0.3% Triton X100, 10% normal donkey serum) for 1 h at room temperature, then incubated overnight at 4°C with primary antibody diluted in blocking buffer. Alexa Fluor-conjugated secondary antibodies were used for detection and samples counterstained with 4^′^6-diamidino-2-phenylindole (DAPI, Sigma). Sections were then mounted on glass slides with coverslips using Fluoro Save (Calbiochem).

### Microscopy

Bright field images were taken and quantified using Lucia imaging software and a Leica FW 4000 upright microscope equipped with a SPOT digital camera. Fluorescence images were obtained using a Leica DM6000 wide field fluorescence microscope equipped with a Leica FX350 camera with x20 and x40 objectives. Images were taken through several z-sections and de-convolved using Leica software. A Leica TCS SP2 confocal laser-scanning microscope was used with x40 and x63 objectives to acquire high-resolution images.

### Image and statistics analysis

All sections for IHC and plasma samples for ELISA were performed in triplicate. Values in the figures are expressed as mean±SEM. A one–way ANOVA was used for data comparison between control and DS. ANOVA was conducted with IBM-SPSS statistic19 software. Differences were considered as statistically significant at *p* < 0.01.

## RESULTS

### Genotype frequency and haplotype co-relation between TREM2, ApoE, Tau, and HLA-DR in DS participants

It was reported that the haplotypes of both *ApoE* and *Tau* have been linked to AD and other neurodegenerative diseases including in frontotemporal dementia, Huntington’s disease, and Parkinson’s disease. We therefore examined alleles and genotype frequencies for *TREM2 (R47H) C/T* mutation, *ApoE* haplotypes (*ApoE,* *ɛ2,* *ɛ3*, and *ɛ4* alleles), *Tau* (rs 9468, tagging *MAPT, H1* versus *H2* haplotypes), and *HLA-DR* (rs3129882 *A/G* polymorphism) by SNPs analysis to determine allele prevalence in DS (*n* = 47, Table 1). The frequency of the *TREM2, R47H C*-allele was (0.95) and *T* allele (0.05) in our DS cohort. Only two DS subjects out of 47 carried the *C/T* mutation, a similar frequency to that previously reported in the sporadic AD population. The frequency of *ApoE* alleles in DS were as follows: *ɛ*2/*ɛ*3 (0.2), *ɛ*3/*ɛ*3 (0.52), and *ɛ*3/*ɛ*4 (0.28, Table 1). The *ɛ*4 frequency was higher in DS compared to control and AD (control frequency: *ɛ*2/*ɛ*3, 0.066; *ɛ*3/*ɛ*3, 0.85, and *ɛ*3/*ɛ*4, 0.08). Thirty-one percent of the DS participants carried one *ApoE* *ɛ*4 allele and none were homozygous; one DS participant (D4, 39 years old, Table 2) with *TREM2 R47H (T* allele) was heterozygous for *ApoE* (*ɛ*3/*ɛ*4). Five years previously, at the first visit, D4 was cognitively normal, but slowly deteriorated and then developed dementia. However, D7 (37 years old) was homozygous for *ApoE* (*ɛ*3/*ɛ*3) and still has no clinical symptoms of dementia yet (Table 2). The frequency of *ɛ*2 alleles in our DS cohort was higher than average (0.11), and interestingly nearly all DS participants above 50 years of age were either carrying *ɛ*3/*ɛ*3 or *ɛ*3/*ɛ*2 haplotypes and had been cognitively normal at first visit (2012) only developing dementia later. The only older DS participant in the cohort (D18, 55 years old male) who carried the *ApoE* *ɛ*3/*ɛ*4 haplotype had dementia from the time of diagnosis at his first visit (Table 2).

**Table 1:** Allele frequency of ApoE, Tau, TREM2 and HLA-DR in DS and control population

**ApoE Allele Frequency**	***ɛ*3/*ɛ*3**	***ɛ*2/*ɛ*3**	***ɛ*3/*ɛ*4**	**p**
Controls (*n* = 50)	0.85	0.066	0.08	≤0.001
DS (*n* = 47, m25, f22)	0.52	0.2	0.28
**Tau SNPs (rs9468)**	**H1/H1**	**H1/H2**	**H2/H2**
Controls (*n* = 50)	0.54	0.33	0.13	≤0.05
DS (*n* = 47)	0.59	0.31	0.09
**TREM2 (R47H)**	**C/C**	**C/T**	**T/T**
Controls (*n* = 50)	1.0	0.0	0.0	≤0.0001
DS (*n* = 47)	0.95	0.05	0.0
**HLA-DR (rs3129882)**	**A/A**	**A/G**	**G/G**
Controls (*n* = 50)	0.49	0.41	0.1	NS
DS (*n* = 47)	0.43	0.43	0.14

**Table 2:** Extended haplotype among DS participants

Number Young	ID N = 19	Sex	Age	Dementia	ApoE	Tau	TREM2	HLA-DR
**1**	**D1**	**M**	**36**	**N**	**E3E4**	**H1/H1**	**C/C**	**A/A ⊗**
**2**	**D2**	**F**	**36**	**N>I**	**E3E4**	**H1/H1**	**C/C**	**A/G**
**3**	**D3**	**F**	**45**	**N>I**	**E3E4**	**H1/H1**	**C/C**	**A/A**
**4**	**D4**	**F**	**39**	**D**	**E3E4**	**H1/H2**	**C/T**	**A/A**
**5**	**D5**	**F**	**47**	**N**	**E3E4**	**H2H2**	**C/C**	**A/A**
**6**	**D6**	**F**	**45**	**N**	**E3E3**	**H1/H1**	**C/C**	**G/G**
**7**	**D7**	**M**	**37**	**N**	**E3E3**	**H2/H2**	**C/T**	**A/G**
**8**	**D8**	**F**	**36**	**N**	**E3E3**	**H1/H2**	**C/C**	**A/A**
**9**	**D9**	**M**	**36**	**N**	**E2E3**	**H1/H1**	**C/C**	**A/G**
**10**	**D10**	**M**	**41**	**N**	**E2E3**	**H1/H2**	**C/C**	**A/G**
**Older**	**N = 27**
**11**	**D11**	**F**	**48**	**N**	**E2E3**	**H2/H2**	**C/C**	G/G*
**12**	**D12**	**M**	**46**	**N**	**E3E3**	**H2/H2**	**C/C**	**A/G**
**13**	**D13**	**F**	**51**	**N**	**E2E3**	**H1/H1**	**C/C**	**G/G**
**15**	**D14**	**F**	**46**	**D**	**E3E4**	**H1/H1**	**C/C**	**A/A**
**16**	**D15**	**M**	**49**	**Died**	**E3E4**	**H1/H1**	**C/C**	**A/A**
**17**	**D16**	**F**	**54**	**D**	**E3E4**	**H1/H1**	**C/C**	**A/A**
**17**	**D17**	**M**	**56**	**P**	**E2E3**	**H1/H1**	**C/C**	**G/G**
**18**	**D18**	**M**	**55**	**D**	**E3E4**	**H1/H1**	**C/C**	**A/G**
**19**	**D19**	**M**	**56**	**D**	**E3E3**	**H1/H1**	**C/C**	**A/A**
**20**	**D20**	**F**	**65**	**D**	**E2E3**	**H1/H2**	**C/C**	**A/A**

N, normal; I, intermediate or mild dementia; D, dementia; P, possibly demented; *green font, protective haplotypes; ⊗red font, high risk haplotypes; blue and black font are common haplotypes. There were 19 young and 27 older DS patients analyzed. Ten different haplotypes were shown from each group.

The *Tau* genotypes, which are found in two forms known as *H1* and *H2* haplotype, and *H1* allele has been reported to be associated with the early-onset AD in the presence of an ApoE *ɛ*4 allele [34]. In DS participants, the frequency of haplotypes were as follows: *H1/ H1* (0.59), *H1/H2* (0.31), and *H2/H2* (0.09) in contrast to that in the control population (0.54, 0.33, and 0.13) (Table 1). *Tau H2* homozygous frequency was lower (0.09) in the DS group compared to the control population. The haplotype frequency of *Tau* and *ApoE* *ɛ4* together was different between those who developed AD dementia before 45 years and those that developed dementia after 45 years of age (*p*≤0.01). Haplotype analysis indicated that individuals with *Tau H1/H1* and *ApoE* *ɛ4* genotype were more prevalent among DS participants (21%) who had an earlier diagnosis of dementia compared to *ApoE* *ɛ4-Tau H1/H2* (6%) and *ApoE* *ɛ4-Tau H2/H2 (2%)* haplotypes (Table 2).

The extended haplotype of DS participants who carried the *TREM2 R47H* mutation was impressive: D7 (male, 37 years old, *ApoE* *ɛ*3/*ɛ*3, *Tau H2/H2, TREM2C/T*, and *HLA-DR A/G*) was non-demented, whereas D4 was (female, 39 years old, carrying *ApoE* *ɛ*3/*ɛ*4, *TauH1/H2, TREM2-C/T,* and *HLA-DR A/A*) developed dementia. Wild-type TREM2 carriers D11 (female, 48 years old was carrying *ApoE* *ɛ2/ɛ3, Tau H2/H2, TREM2C/C, and HLA-DR G/G*) and D12 (male, 46 years old was carrying *ApoE* *ɛ3/ɛ3 Tau H2/H2, TREM2C/C,* and *HLA-DRA/G*) had not developed dementia, indicating that *H2* homozygosity could be protective in DS subjects (Table 2).

The *HLA-DR*, *G* allele frequency was slightly higher but non-significantly in DS (control frequency 0.49, and in DS 0.56, Table 1). We have identified two **extended haplotypes** in DS cohorts which are the *TREM2-C, HLA-DR-G, Tau-H2,* and *ApoE-ɛ2* variants as being **a protective haplotype *(C-G-H2-ɛ2)*** and a high-risk or disease haplotype, the *TREM2-T, HLA-DR-A, Tau-H1,* and *ApoE-ɛ4 (T-A-H1-ɛ4)* (Table 2). Unfortunately, the number of DS samples available for this study was too small to infer any statistical significance (*n* = 47). This analysis would be interesting to be repeated with a larger cohort.

### ApoE protein level was haplotype dependent and soluble TREM2 and Aβ_42_ protein levels were age dependent and declined in DS with dementia progression

After identifying informative allelic effects, we investigated whether there was any correlation between *TREM2, Tau*, and *ApoE* allele frequency and plasma protein concentration? We measured the plasma protein levels of TREM2, ApoE, tau, and Aβ_42_ and Aβ_40_ in DS and age-matched controls using ELISA. We have divided DS subjects into two age groups: a younger DS group (*n* = 19, age between 30–44 years), referred as Y-DS, and an older DS group referred as O-DS (*n* = 27, age between 45–70 years).

**Fig.1:**
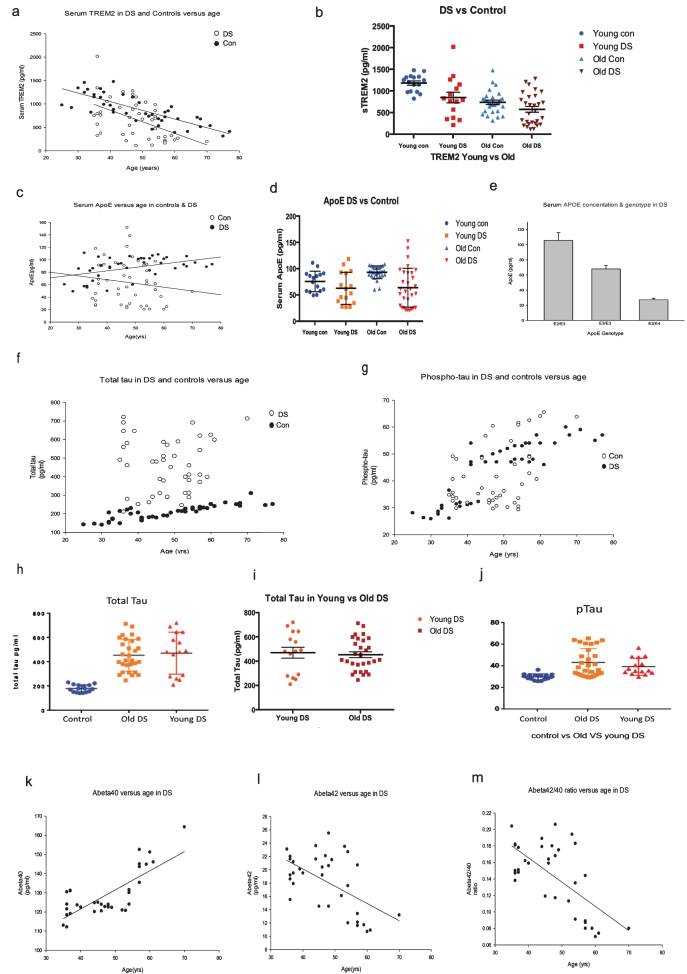
Plasma ApoE, soluble TREM, and Aβ_42_ protein levels were declined in DS with dementia progression. Plasma protein levels of TREM2, ApoE, tau, Aβ_42_, and Aβ_40_ in DS and age-matched controls were determined by ELISA. Levels of sTREM2 were found to be significantly higher in controls compared to DS; R^2^ = 0.78, *p*≤0.0001 (a). Soluble TREM2 level is age dependent, decreasing with age and disease progression in DS. Horizontal scattered bars indicate median sTREM2 concentration per group (b). The levels of ApoE was widely distributed in DS, R^2^ = 0.44, *p*≤0.001 (c). ApoE plasma levels were higher in older controls compared to age-matched older DS as shown by scattered plot (d). *ApoE* *ɛ2* carriers have higher ApoE levels than *ApoE* *ɛ4* heterozygous subjects, R^2^ = 0.78, *p*≤0.0001 (e). The total tau (t-Tau) level was not significantly different between Y-DS compared to O-DS, R^2^ = 0.38, *p*≤0.01 (f) and shown by scattered plot (h, i). Whereas p-tau was significantly higher in O-DS subjects compared to Y-DS, R^2^ = 0.45, *p*≤0.001 (g), and as shown by scattered plot (j). The plasma Aβ_40_ level was found to be increased with age, R^2^ = 0.77, *p*≤0.0001 (k), and Aβ_42_ level was decreased with age, R^2^ = 0.56, *p*≤0.0001 (l). The Aβ_42_/Aβ_40_ ratio, R^2^ = 0.66, *p*≤0.0001, decreased with age and dementia progression in DS (m).

sTREM2 levels were found to be significantly higher in controls compared to DS (Fig. 1a, R^2^ = 0.78, *p*≤0.0001). Furthermore, sTREM2 levels were higher in Y-DS than O-DS (horizontal bars indicate median sTREM2 concentration per group, Fig. 1b). This data is in agreement with our previous findings that TREM2 serum protein levels are age dependent and decrease with age and disease progression in DS [18].

ApoE plasma levels were then measured in the same samples by ELISA. The levels of ApoE were widely distributed in DS (R^2^ = 0.44, *p*≤0.001, Fig. 1c). ApoE plasma levels were higher in older controls compared to age-matched older DS (Fig. 1d). Although we have *ApoE* genotype information of the DS participants, it was not available for the control subjects. Surprisingly, Y-DS subjects carrying an *ApoE* *ɛ2* genotype had the highest plasma ApoE (range between 118.2–188.5 pg/ml) compared to O-DS (range between 49.1–97.7 pg/ml, *p*≤0.001, Fig. 1d). In contrast, among the *ApoE* *ɛ4* carriers, levels were comparatively lower in O-DS (range from 20.4–25.7 pg/ml) than in Y-DS (range 22.7–44.5 pg/ml, Fig. 1e). These data indicate that ApoE plasma protein level is haplotype dependent, where *ApoE* *ɛ2* heterozygous carriers have higher ApoE levels than ApoE*ɛ*4 heterozygous carriers (R = 0.78, *p*≤0.0001, Fig. 1e).

We then measured t-Tau and p-Tau levels in plasma of Y-DS versus O-DS participants (Fig. 1f, g). The t-Tau levels were not significantly different between the Y-DS (range 210.6–720.4 pg/ml, Fig. 1f) and O-DS (range 240.8–712.6 pg/ml, R^2^ = 0.38, *p*≤0.01, Fig. 1f, h, and i), whereas p-Tau was significantly higher in O-DS participants (range 29.3–75.5 pg/ml) compared to Y-DS (range 29.8–56.7 pg/ml, R^2^ = 0.45, *p*≤0.001, Fig. 1g, j). Plasma Aβ_40_ levels were found to increase with age, in Y-DS the range was 113.0–131.2 pg/ml and in O-DS the range was 120.5–164.3 pg/ml (R^2^ = 0.77, *p*≤0.0001, Fig. 1k), whereas Aβ_42_ was 15.5–23.1 pg/ml in Y-DS and in O-DS it was, 10.7–22.1 pg/ml (R^2^ = 0.56, *p*≤0.0001, Fig. 1l). Surprisingly, plasma Aβ_42_ level decreased with age in DS participants (Fig. 1k, l). Additionally, the Aβ_42_/Aβ_40_ ratio decreased with age and disease progression in DS (0.12–0.18 in Y-DS compared to 0.07–0.20 in O-DS (R^2^ = 0.66, *p*≤0.0001, Fig. 1m). Among the Y-DS participants (aged between 30–44 years), 36% of subjects who were dementia-free at time of diagnosis had developed dementia within five years. In the O-DS group (aged between 45–70 years), 26% participants were Aβ-positive as measured with PET-PiB (PET-[^11^C]-Pittsburgh Compound-B) and had early dementia. After five years, 52% of these O-DS participants had developed dementia.

### TREM2 and tau proteins did not co-localize with Aβ_42_ senile plaques

**Fig.2:**
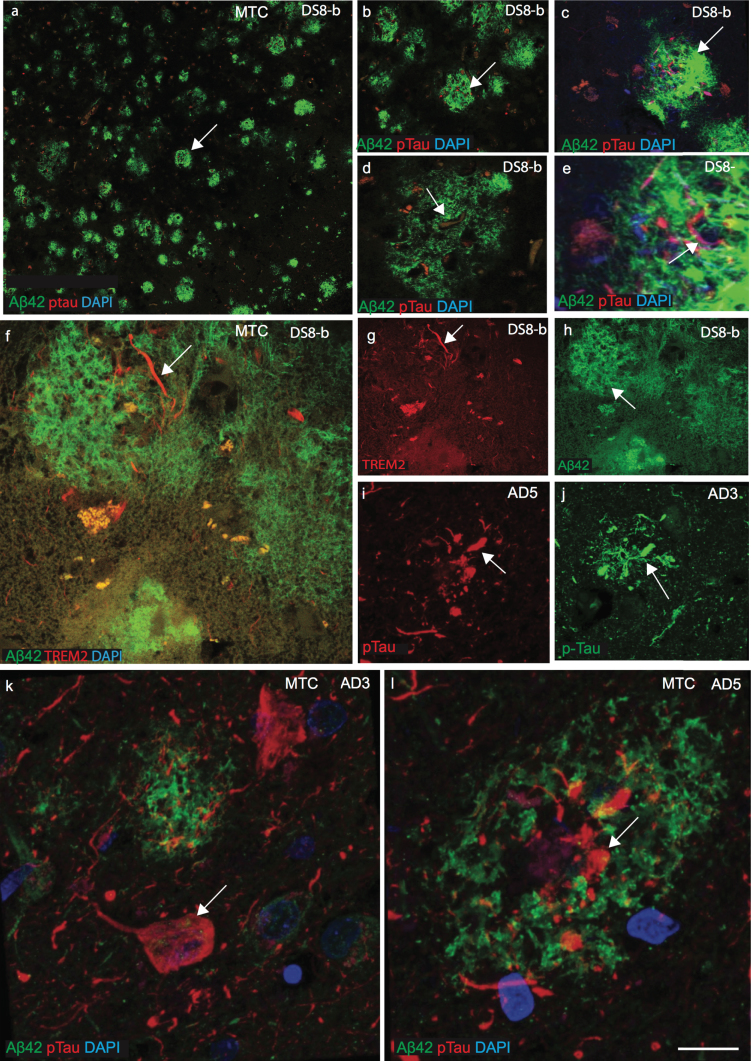
**TREM2 and tau proteins did not co-localize with Aβ_42_ senile plaques in mid temporal cortex.** Double immunofluorescence staining and confocal images were performed on the mid temporal cortex (MTC) of AD and DS brain sections using rabbit monoclonal anti-Aβ_42_ (rab-mAb Aβ_42_, green) and mAb anti-phospho-tau (AT8, red) antibody, DAPI for nuclear staining (Blue). Aβ_42_ immunoreactivity was visible in the senile plaques (SPs) but did not co-localize with p-Tau (a–e). In DS brain (DS8), the layer III of MTC stained for anti-Aβ_42_ (green) and showed many mature plaques (f and h). TREM2 was only present around the SPs and visible in the neuropil thread (f and g). Further staining of different cases of AD (AD3 and AD5, Supplementary Table 1) and images was captured by confocal microscopy, showed that mature Aβ_42_ positive SPs did not co-localize with p-Tau positive neurofibrillary tangles. Scale bar in a–c = 50 μm, d-k = 25 μm, and l = 10 μm.

To investigate whether there is any influence of plasma protein in the periphery on protein expression in the brain parenchyma, the cellular location of TREM2, tau, ApoE, and Aβ_42_ was examined by immunofluorescence staining on postmortem brains sections from the superior frontal cortex (SFC), hippocampus (HP), and mid temporal cortex (MTC) of AD, DS, and age-matched controls (*n* = 10 in each group: aged between 46 to 76 years, Supplementary Table 1). Confocal images of DS MTC brain sections showed many mature SPs with well-defined structures of amyloid protein positive depositions (stained with Aβ_42_, antibody) but which did not co-localize with p-Tau (stained with AT8, antibody Ser202/Thr 205 epitope; see Fig. 2a-e). In DS brains, Aβ_42_ positive plaques appeared considerably more dense compared to age-matched AD brains (Fig. 2a-f). In AD brains, AT8 positive p-Tau was visible in the center of plaques but it did not appear to co-localize with Aβ_42_ positive SP (Fig. 2d, e). Confocal images from AD and DS brain showed that both TREM2 and p-Tau were visible in NTs and in NPs (Fig. 2f, g, i, and j). However, in AD brain sections, p-Tau positive NFT were visible in the periphery of plaques or in the core of SPs (Fig. 1k, l). We previously reported that TREM2 protein was present in the surviving neurons and here we show that it is present in the NTs and the NPs but it was not found within NFTs in the DS brains [18].

### Increased insoluble tau in the hippocampus and in entorhinal cortex of DS brain

**Fig.3:**
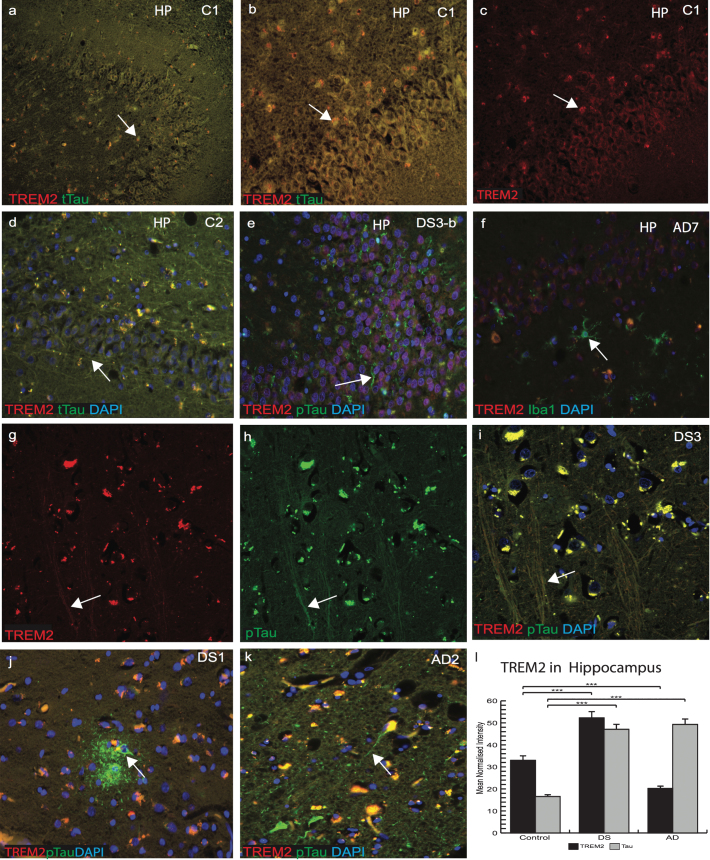
**Increased insoluble tau in the hippocampus and in entorhinal cortex of DS brains.** Confocal microscopy analysis was performed on controls, AD, and DS brain sections particularly in the hippocampus (HP) and entorhinal cortex. Total anti-Tau (HT7) for control brains and anti-p-Tau (AT8) for AD and DS were used and DAPI for nuclear staining. In a young control brain (C1, Supplementary Table 1), t-Tau (recognized by HT7, green) and TREM2 (red) co-localized in the pyramidal neurons of HP, in dentate gyrus granule cells, subiculum, cortical, and subcortical areas (a–d). In a younger DS subject (DS3-b, Braak stage 2, Supplementary Table 1), TREM2 was visible in the dentate gyrus granule cells and insoluble p-Tau in the pyramidal neurons (in the axons) with limited co-localization (e-g). In AD brains, limited TREM2 expression was seen in the HP and did not co-localized with Iba1 positive activated microglia (f). Confocal images of a DS brain (DS3), TREM2, and p-Tau co-localized in the axons (g-i), but TREM2 was not visible in neurofibrillary tangles (j). In an AD brain section (AD2), TREM2 proteins were present in the cell body, whereas p-Tau was visible in the neuritic threads, neurofibrillary tangles, and damaged axons (k). TREM2 levels were highest in controls and lowest in AD brains (l, *p*≤0.0001). Scale bar in a–f = 50 μm, g–k = 25 μm. Image intensity measured by Image J. Error bars indicate SEM, ^**^p < 0.001, ^***^p < 0.0001.

We then further explored the association and expression of TREM2 and tau in the DS brain with particular focus in the HP and entorhinal cortex. Sections of control, DS, and AD brains (*n* = 10 from each group) were stained with anti-t-Tau (t-Tau, HT7 antibody) for control brains and anti-p-Tau (AT8) for AD and DS brains. In a young control brain (C1, 45 years old, Supplementary Table 1), t-Tau (t-Tau, recognized by antibody HT7) and TREM2 co-localized in the pyramidal neurons of the HP, CA1, dentate gyrus granule cells, subiculum, cortical, and subcortical areas (Fig. 3a–c). In another control (C2, 45 years old), t-Tau was present in pyramidal neurons of CA1, CA3, and in the neurofilaments of subiculum (Fig. 3d). In a younger DS subject (DS3-b, 46 years old, with Braak stage 2, the youngest DS subject in this cohort), TREM2 staining was visible in the dentate gyrus granule cells. Insoluble p-Tau was seen in the axonal pyramidal neurons with limited co-localization with TREM2 (Fig. 3e). Compared to an AD case (AD7, 88 years old, Supplementary Table 1), where most of the granule cells were damaged, Iba1 positive (microglial marker) cells were present close to the damaged neurons but minimal TREM2 expression was seen (Fig. 3f). Confocal analysis of the DS3-b brain showed very limited TREM2 co-localization with p-Tau in NTs (Fig. 3g-i). In an older DS brain from the MTC (DS1-b, 56 years of age, Braak stage 6), p-Tau positive mature SPs and NFTs were observed but TREM2 was only visible in partially damaged neurons in the surrounding area and did not co-localize with p-Tau positive cells (Fig. 3j). In another AD (AD2, 88 years old) brain section, both proteins were present in the cell body whereas p-Tau was visible in the NT, NFT, and damaged axons (Fig. 3k). Note that TREM2 levels were highest in control and lowest in AD (Fig. 3l, *p*≤0.001).

### TREM2 expression was reduced in white matter tracts in DS brain

**Fig.4:**
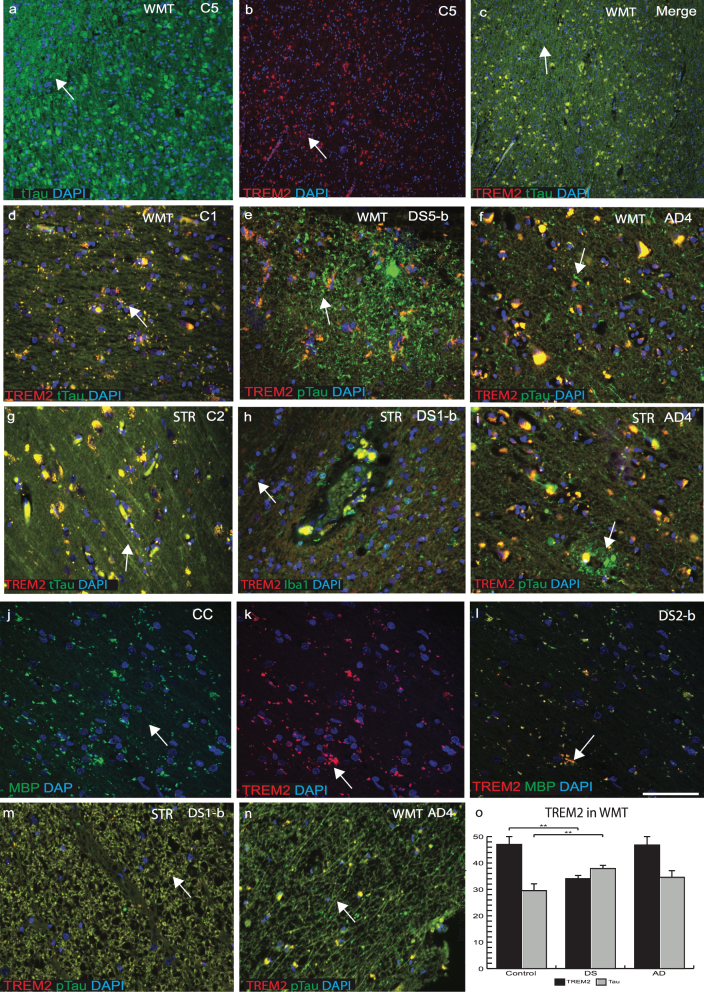
**TREM2 expression was reduced in white matter tracts in DS brains.** White matter tracts (WMTs) are narrowed in DS brains as shown by PET imaging data. Thus, WMTs and corpus callosum (CC) of control, AD, and DS brain sections were analyzed by immunofluorescence using TREM2, t-Tau, or p-Tau antibodies. In control brains in the superior frontal cortex (grey matter), TREM2 (red) and t-Tau (HT7, green) co-localized in cortical neurons (a–c), whereas in striatum (STR), WM-tract both proteins were present in oligodendrocytes (d). In DS and AD brains, p-Tau (stained with AT8) were present in the in the neuropils and in neurofibrillary tangles and did not co-localize with TREM2 (e and f). In control brains, t-Tau protein was present in the WMT of STR and in the CC (g). In DS brains in STR, WMT was damaged and high infiltration of Iba1 positive microglial cells were visible (h). TREM2 did not co-localize with p-Tau positive SP in AD brain sections (i). Confocal image of DS brain, WMTs of the CC were shrunken (atrophic), and some MBP positive oligodendrocytes were co-localized with TREM2 (j-l). Both proteins (TREM2 and p-Tau) were detected in WMT in DS and AD cases but with reduced expression in DS (m-n). TREM2 levels were higher in WMT of control and AD compared to DS (o, *p*≤0.001). Scale bar in a–c = 75 μm, d–i, m, *n* = 25 μm, j–l = 20 μm. Image intensity measured by Image J. Error bars indicate SEM, ^**^p < 0.001.

Our PET image analysis data indicated that there was narrowing of white matter tracts (WMT) and enlargement of putamen volume in DS brain [31]. We therefore analyzed WMT in SFC, striatum (STR) and corpus callosum (CC) of control, AD, and DS brains with anti-TREM2, and anti-t-Tau (HT7) or anti-p-Tau (AT8). In control brains, in white matter (WM) and SFC gray matter, TREM2 and t-Tau co-localized in cortical neurons, particularly in the axons (Fig. 4a–c). In DS brain sections, p-Tau and limited TREM2 were present in the WM of cortex and in STR (Fig. 4e, h). p-Tau expression was found to be higher in NTs than in NFTs in DS brains and it had limited co-localization with TREM2 (Fig. 4e). Similarly, in AD brain, particularly in WMT and in STR, large numbers of p-Tau positive NTs and NFTs were visible (Fig. 4f & i). In control brains, t-Tau protein was present in the WMT of STR and in the CC, with strong co-localization with TREM2 and t-Tau (Fig. 4g). Furthermore, in the DS brain, WMT was damaged and there was a high degree of infiltration with of Iba1 positive microglia (Fig. 4h). Additionally, in the DS brain tissue, WMTs of the CC were shrunken (atrophic), and some MBP (mature oligodendrocyte marker) positive oligodendrocytes present were positive for TREM2 (Fig. 4j–l). Notably, both proteins (TREM2 and tau) were detected in WMT in controls and AD cases but with a reduced expression in DS, indicating WM damage in DS brain may involve atrophy (Fig. 4j–i, m, and n). TREM2 levels were higher in WMT of control and AD compared to DS brains (Fig. 4o, *p*≤0.001).

### p-Tau aggregates and ApoE were visible in the fenestrated capillaries of choroid plexus and sTREM2 in macrophages

**Fig.5:**
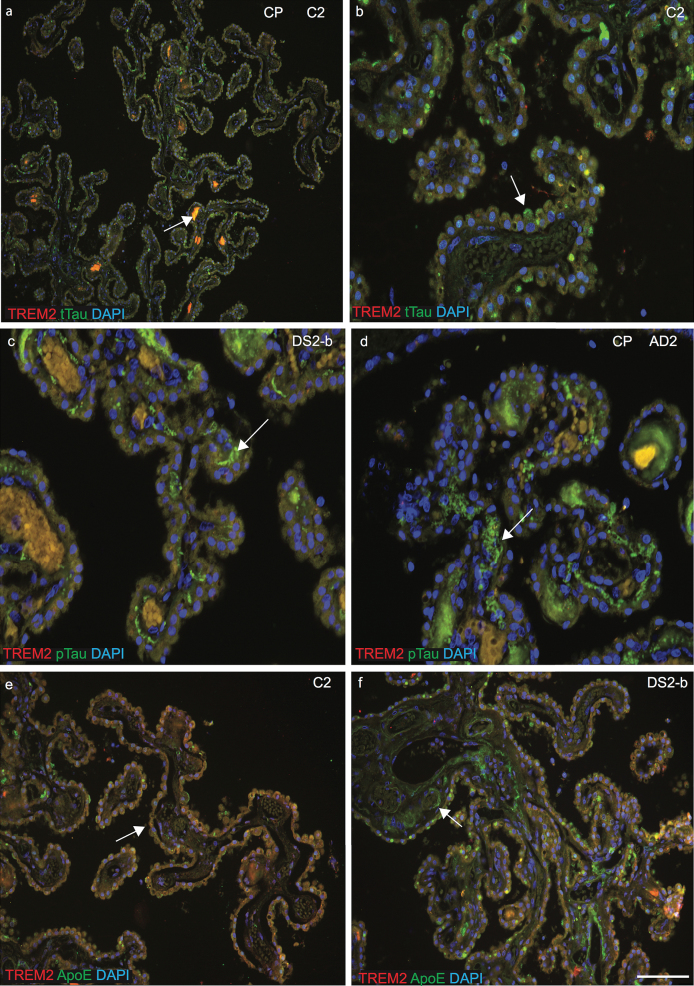
**Phospho-tau aggregates and ApoE were visible in the fenestrated capillaries of choroid plexus and sTREM2 in the macrophages.** Brain sections containing choroid plexus (CP) in the lateral ventricles and third ventricles from control, AD, and DS subjects were stained with anti-TREM2 and anti-Tau (t-Tau or p-Tau), or anti-TREM2 and anti-ApoE antibodies and analyzed by confocal microscopy. DAPI was used for nuclear staining. In control brain, both TREM2 and anti-t-Tau were present in CP cuboidal epithelial cells (CPE) surrounding a core of fenestrated capillaries and connective tissues (a). Some soluble t-Tau protein was visible inside fenestrated capillaries and TREM2 was visible in stromal capillary (vesicles) and in the stromal macrophages (a and b). In DS and AD brain dense fibrillary p-Tau present in psammoma bodies (calcified intracellular inclusion structures), was visible in the stroma and TREM2 co-localized in the vesicles and in stromal macrophages (c and d). In control CP, both proteins TREM2 (red) and ApoE (green) were visible in CPE cells and in stromal macrophages that appeared normal with healthy structure (e). In contrast, in DS brains ApoE was visible in the “amyloid biondi” bodies (complex filamentous ring-like structures associated with lipid droplets, showing with an arrow) and lipofuscin (yellow or brown intracellular structures composed of lipid molecules (f). Scale bar in a = 50 μm, b–f = 25 μm.

As previously, we have found that the CP is damaged in AD brain and in an AD mouse model and as CP is a conduit between the peripheral circulation and central nervous system via the CSF, we extended our investigation to the CP in DS brain [26]. The CP is a complex structure, which hangs inside the ventricles of the brain and consists mainly of CPE cells surrounding fenestrated capillaries. CSF is formed by the CPE and many soluble proteins can enter the brain parenchyma via the CP [35]. Brain sections containing CP in lateral ventricles and third ventricles from control, AD, and DS tissues were stained with anti-TREM2 and anti-tau or anti-TREM2 and anti-ApoE antibodies. In control brains, both TREM2 and anti-t-Tau were present in CP cuboidal epithelial cells surrounding a core of fenestrated capillaries and connective tissues (Fig. 5a). Some soluble t-Tau protein was visible inside fenestrated capillaries and TREM2 was visible in stromal capillary (vesicles) and in the stromal macrophages (Fig. 5a, b). In DS and AD brain, dense fibrillary p-Tau was present in “psammoma” bodies (calcified intracellular inclusion structures), and in the stroma and TREM2 co-localized in the vesicles and in stromal macrophages (Fig. 5c, d). Similarly, in controls, both the TREM2 and ApoE protein were visible in CPE cells and in stromal macrophages that appeared normal and healthy in structure (Fig. 5e), whereas in DS brains, ApoE was visible in the “Amyloid Biondi” bodies (complex filamentous ring-like structures associated with lipid droplets) and lipofuscin (yellow or brown intracellular structures composed of lipid molecules (Fig. 5f). In AD brains, epithelial membranes of the CP were damaged and distorted, and fibrillary p-Tau occupied fenestrated capillaries that could potentially block the Aβ clearance process (Fig. 5d). These finding indicated that the CP-CSF system exhibits morphological changes and a functional decline with aging, in DS and in AD brain.

## DISCUSSION

The most common form of DS is due to trisomy 21, and many neurobiological studies in older DS participants have focused primarily on AβPP processing and the temporal events that are thought to lead to Aβ pathogenesis [36, 37]. In addition to Aβ accumulation, middle-aged individuals with DS develop NFTs, cerebrovascular pathology (cerebral amyloid angiopathy), WM pathology, oxidative damage, neuroinflammation, and neuronal loss [38]. Aβ accumulation is a very early event in the DS brain, that includes formation of soluble Aβ species with different conformations of peptide such as oligomers, protofibrils, and Aβ-derived diffusible ligands, visible inside the cells, particularly in the neurons [39]. Aβ_40_ and Aβ_42_ have different properties for example: Aβ_40_ being more rapidly degraded within the lysosomes than the more toxic Aβ_42/43_ species [40]. The amount of Aβ in the brain is also determined by its clearance from the brain.

Apolipoprotein E (ApoE) is an important protein that influences the clearance and metabolism of Aβ from brain parenchyma [41]. It has also been reported that *ApoE* alleles modulate chronic inflammation and some aspects of aging in brains and arteries [42]. Blood ApoE mediates the clearance of triglyceride and cholesterol-rich lipoprotein components, and brain ApoE transports cholesterol to neurons [43]. In general, the *ApoE* *ɛ*4 allele shortens lifespan by several years and accelerates degenerative changes in arteries and brains [44]. The presence of an *ApoE* *ɛ*4 allele doubles the cerebral amyloid plaque burden and shortens lifespan in DS [45]. Furthermore, neuroinflammation, including the brain’s innate immune response, is also recognized as a key component of neurodegenerative disorders. In AD and DS, neuroinflammation has been linked to both the exacerbation of amyloid plaques and NFTs in addition to increased clearance of amyloid plaques. The discovery of the R47H mutation in the *TREM2* gene in AD and DS established a closer link with immunity and neuroinflammation [18].

In this paper, we initially analyzed genotype and haplotypic associations between all four genes (*Tau, TREM2, ApoE,* and *HLA-DR*) and identified a “disease specific” and a “neuroprotective” haplotype. The *Tau* gene located on human chromosome 17q21.31 and a 900 Kb inversion in *Tau* gene created *H1* (the non-inverted sequence) and *H2* (the inverted sequence) haplotypes. The *H1* haplotype is a risk factor locus for progressive supranuclear palsy, corticobasal degeneration, Lewy body dementia, and Parkinson’s disease [46]. In this paper in the DS cohort, we have reported that individuals with a *Tau H1/H1* and *ApoE* *ɛ4* genotype were more prevalent among DS participants (21%) who had an earlier diagnosis of dementia compared to *ApoE* *ɛ4* and *Tau H1/H2* haplotypes (6%). This data may indicates that *Tau H2* homozygosity could be protective in DS participants, as was previously reported in AD [47].

As we reported, only two DS participants out of 47 who carried the *TREM2-C/T* mutation, and a similar frequency was previously reported in the AD population [48]. One DS participant (D4, 39 years old) with *TREM2 R47H (T* allele) who was heterozygous for *ApoE* (*ɛ*3/*ɛ*4) slowly deteriorated and developed dementia, whereas another DS participant (D7, 37 years old) who was homozygous for *ApoE* (*ɛ*3/*ɛ*3) but had no clinical symptoms of dementia as yet. The *ApoE* *ɛ*2 allele has been associated with a decreased risk of AD [49]. The frequency of *ɛ*2 alleles in DS was higher (20%), and interestingly nearly all DS participants above 50 years of age who were either carrying *ɛ*3/*ɛ*3 or *ɛ*3/*ɛ*2 haplotypes were cognitively normal or developed dementia at a much later stage. There was a correlation between *TREM2-T, HLA-DR-A, Tau-H1,* and *ApoE*-*ɛ*4 (*T-A-H1-ɛ4*) and a high-risk of dementia in contrast to *TREM2-C, HLA-DR-G, Tau-H2*, and *ApoE*-*ɛ*2 haplotype, which was protective (*C-G-H2-ɛ2*).

To determine plasma protein level in DS, we investigated TREM2, Aβ_42_, Aβ_40_, tau, and ApoE in DS and age-matched controls using ELISA. Soluble TREM2 protein levels declined and p-Tau level increased in DS serum with age and dementia progression. We found that the effect of *ApoE* allele is isoform specific in DS. The Y-DS participants carrying *ApoE* *ɛ*2 genotype had the highest plasma levels compared to the O-DS participants. In contrast, those with the *ApoE* *ɛ*4 allele, the ApoE plasma levels were comparatively lower in the O-DS than in the Y-DS. These data indicated that ApoE protein levels were haplotype dependent as were effects with age. *ApoE* *ɛ*2 heterozygous DS carriers have higher ApoE serum protein levels than *ApoE* *ɛ*4 heterozygous. The majority (∼75%) of plasma ApoE is produced by hepatocytes, creating a hepatic pool that is important for lipid metabolism, while the second most common site of synthesis is the brain particularly in the glial cells (astrocytes and microglia) [43]. The production of different amounts of ApoE protein due to different ApoE isoforms are likely to be due to their amino-acid residue variation impacting the protein structure. *ApoE* *ɛ*2 carriers have two cysteines in 112 and 158 position, in *ApoE* *ɛ*3 cysteine 158 is replaced by an arginine whereas in *ApoE* *ɛ*4 both cysteines are replaced by arginines [43]. The changes of both cysteines to arginines affects the helix structure and binding capacity of many lipoproteins and cholesterols due to Cys-Cys binding with many heme and iron proteins (Raha-Chowdhury, unpublished data). In addition to the effects on fibrillogenesis, ApoE is an Aβ chaperone, promoting transport across the blood-brain barrier, a process that is known to be impaired in AD as a consequence of vascular damage [50]. There are *ApoE* isoform-dependent differences in the anti-inflammatory role in neurodegenerative diseases and such differences might in part explain the differential risk for AD caused by *ApoE* isoforms. In support of this hypothesis, several studies demonstrated that exogenously applied *ApoE* *ɛ4* has a more robust proinflammatory activity than *ApoE* *ɛ3* in astrocytes and microglial cells [51]. Besides influencing brain aging, *ApoE* alleles also affect brain development. Cortical neurons of young transgenic-ApoE *ɛ4* overexpressing mice have less dendritic complexity, which may be a factor in their impaired spatial memory [52].

It is still debatable whether plasma or serum Aβ species can serve as reliable biomarkers for diagnosis dementia or its prodromal stage in DS. According to a large-scale meta-analysis in sporadic AD, plasma t-Tau was the only blood biomarker with a sufficient effect size [53]. To our knowledge, except for one study, plasma p-Tau and t-Tau has been little investigated in DS [54]. Plasma Aβ species have been expected to discriminate between demented and non-demented individuals with DS and many studies measuring plasma Aβ species in DS agreed that plasma levels of Aβ species in DS were significantly higher than those in normal individuals, mainly because of overproduction of the AβPP [55]. Our aim in this study was to assess and compare plasma levels of p-Tau and t-Tau with other blood biomarkers including plasma Aβ in adults with DS and to compare with an age-matched control population. We found that reproducible measurements of plasma p-Tau were difficult to measure because of its very low concentration in peripheral blood. We found that the plasma Aβ_40_ level was increased in the O-DS group compared to the Y-DS and Aβ_42_ levels decreased with age in DS participants (R^2^ = 0.56, *p*≤0.0001) as reported previously in AD in the typically developing population. Among Y-DS group (aged between 30–44 years) there was a higher frequency of *ApoE* *ɛ*4 and 36% of participants who were non-demented at baseline had developed dementia within five years. In the O-DS subjects (aged between 45–70 years), 26% of participants who were PiB positive and who had no dementia, but after five years 52% of these O-DS participants had developed dementia. To our knowledge, this paper shows for the first time a correlation between plasma proteins and clinical phenotype changes in 47 adults DS who underwent structural and amyloid PiB imaging and in whom putative blood biomarkers identified a high-risk group.

For cellular localization, we have examined postmortem DS, AD and control brain tissues (*n* = 10 in each group) by immunohistochemistry and analyzed by bright field or confocal microscopy, using TREM2, tau (t-Tau and p-Tau), Aβ_40_, Aβ_42_, and ApoE antibodies. Although many published papers have reported TREM2 protein expression in microglia [56, 57], we have shown for the first time that TREM2 protein is expressed in human DS brains particularly in the cortical and in HP neurons. Our previous work showed that TREM2 mRNA was not synthesized in the neurons, but it could be entering the brain parenchyma and in the neuronal cells as soluble TREM2 entirely from the periphery and that it is involved in neuroplasticity [15]. In postmortem brain sections of DS and AD, we showed that TREM2 and Aβ_42_ were located in surviving neurons very close to SPs in frontal and entorhinal cortices. We have observed that, in young DS brains, Aβ_42_ positive dense Aβ accumulation (as cotton wool shaped) were present and in some locations, particularly in SFC and in MTC, and in older DS brain tissue these plaques may progress in to fibrillary SPs. Tau protein was visible in the periphery of dystrophic neurites and of NFTs in AD brain. With disease progression, large swollen dystrophic neurites became NFT and no TREM2 expression was seen to be associated with Aβ_42_ positive SPs or NFTs. Cotton wool shaped plaques were present in DS brain in the absence of NFTs, which may be due to the excess Aβ accumulation as a result of the gene triplication effect of AβPP on Ch21. Although a large amount of Aβ (Aβ_40_) exists in a soluble form, insoluble deposits also begins to progressively form over time. In DS, Aβ accumulates in the neurons (intracellular), and later as amyloid deposition. As deposition increases, the Aβ_42_/Aβ_40_ ratio decreases leading to the formation of extracellular (Aβ_42_) SPs. It is also apparent that diffuse plaques precede neuritic plaques with age in the cortex of DS subjects.

In the HP of normal brain, TREM2 and t-Tau protein were visible in granule cells of the dentate gyrus, supporting our recent publication regarding TREM2’s involvement in neurogenesis [15]. Furthermore, the presence of TREM2 in axons and dendritic processes of young DS brains and its co-localization with p-Tau in NTs supports its role as a soluble transporter. In brain tissue obtained at postmortem from younger people with DS, we observed AT8 positive p-Tau in the molecular layer of HP, which was followed later by NFT in the HP CA1 region and subiculum, and neuronal loss in the entorhinal cortex. In older DS brain tissue, p-Tau was visible in the NFTs but without the expression of TREM2, suggesting the presence of TREM2 protein only in the unaffected neurons. In younger DS brain, a large amount of t-Tau exists in a soluble form, and insoluble p-Tau begins to accumulate progressively over time.

TREM2 expresses in oligodendrocytes in the WMT, the olfactory bulb, CC, and STR bundles, thus indicating that TREM2 protein may have a role in myelination [15]. The loss of functional TREM2 could be a contributing mechanism in demyelination deficits seen in DS and which would, in turn, increase the susceptibility for neuronal loss [58]. Myelination defects are one of the features of DS children that may continue into adulthood with the potential to accelerate neuronal loss and premature ageing. Brain sections from DS, AD, and control cortex, the CC and STR were stained with anti-TREM2 and anti-t-Tau or anti-p-Tau (AT8). In control brains, t-Tau protein was present in the WMT of STR and in the CC with strong co-localization with TREM2. In contrast in DS brains, STR was damaged and a high infiltration of Iba1 positive microglia was visible, the WMTs of the CC were shrunken (atrophic) and some MBP positive oligodendrocytes (mature oligodendrocyte marker) were positive for TREM2. Notably, both proteins (TREM2 and tau) were detected in WMT in controls and AD cases but with a reduced expression in DS brain, indicating a role of TREM2 in axonal transport with tau that could be impaired due to WM defect in DS.

The CP plays a pivotal role in brain homeostasis. Indeed, the structure, function and location of the CP-CSF system has a significant influence on physiological and pathological CNS functioning [59]. It is the main source of CSF production and is responsible for the removal of toxic waste products. The CP may also be regarded as a “gatekeeper” of the brain given the presence of resident inflammatory cells [60]. In normal control brain, TREM2 protein was highly expressed in the CP epithelial cells and particularly in the stromal macrophages and close to the ventricles and subarachnoid space. In DS and AD brains dense fibrillary p-Tau present in “psammoma bodies” (calcified intracellular inclusion structures), was visible in the stroma, whereas in DS brains ApoE was visible in the “amyloid biondi” bodies (complex filamentous ring-like structures associated with lipid droplets) and lipofuscin (yellow or brown intracellular structures composed of lipid molecules). Both in AD and DS, CPE cell functionality is heavily affected as is reflected by the loss of structural integrity and changes in secretory activity. These might play a central role in disease initiation or exacerbation or maintenance of disease progression [61].

Many other immune molecules are located in the extended HLA cluster (TREM2, TREMEL1, HLA-DR, 1C7, and C1Q). These proteins could have particular roles in different types of immune cells and confer immunoprotective abilities [62]. The mechanism by which an extra chromosome 21 produces abnormalities in the immune system seen in ageing in people with DS, including susceptibility to autoimmune diseases and recurrent infections, are still not fully understood. The foundation for premature ageing in DS may be in part due to the triplication and over expression of AβPP, which is encoded on chromosome 21. Other genes may also play a role in tau phosphorylation in DS brain as Aβ_42_ upregulates both DYRK1A [63] and RCAN1 [64], but this needs further evaluation. Thus, overexpression of all three genes in DS may co-operate to drive tau pathology and neurodegeneration.

### Conclusion

Although Aβ overexpression in the brain is the key gene defect in DS, three other genes, *Tau, ApoE*, and *TREM2*, also could have a distinct role in Aβ clearance and neuroprotection. A vast amount of Aβ enters in the brain from periphery via blood and to be carried by astrocytes and macrophages. In the initial stages of the diseases processes, large numbers of activated astrocytes and microglia were involved in the clearance processes to prevent plaques formation, but with the failure of that function Aβ becomes trapped in the SPs. Tau protein transport may be ApoE and other lipoproteins dependent. We showed that ApoE protein levels were haplotype dependent. The major source of ApoE protein is synthesized in the liver and carried via serum, entering the brain parenchyma via CP epithelial cells and blood vessels, followed by being taken up by astrocytes and other glia cells. TREM2 protein enters via stromal macrophages of CP and microglia as described above. All four proteins orchestrated the task of protecting the brain from neurodegeneration. Pharmacological targeting of TREM2 to suppress the inflammatory response and modifying ApoE *ɛ*4 isoform via CRISPA/cas9 may provide a new approach for developing therapeutic strategies in the treatment of neuroinflammation and other neurodegenerative diseases.

## Supplementary Material

Supplementary Material
