# Supplementary material for: Choroid Plexus Acts as Gatekeeper for TREM2, Abnormal Accumulation of ApoE, and Fibrillary Tau in Alzheimer’s Disease and in Down Syndrome Dementia
Source: J Alzheimers Dis. 2019 May 7;69(1):91–109. doi: 10.3233/JAD-181179 (PMC6598012; doi:10.3233/JAD-181179)
Supplement: Supplementary Material [file jad-69-jad181179-s001.docx]

**Supplementary Material**

**Choroid Plexus Acts as Gatekeeper for TREM2, Abnormal Accumulation of ApoE, and Fibrillary Tau in Alzheimer’s Disease and in Down Syndrome Dementia**

**Supplementary Table 1.**

Human brain samples from Down’s syndrome (DS), Alzheimer’s disease (AD), and age-matched controls analyzed in this study.

| Case number | Category | Age | Gender | PM delay (h) | Braak stage | Cause of death |
| --- | --- | --- | --- | --- | --- | --- |
| **Down’s Syndrome** |  |  |  |  |  |  |
| DS1 | DS | 56 | F | 6 | 6 | Not known |
| DS2 | DS | 76 | F | 18 | 6 | septicemia |
| DS3 | DS | 46 | F | 8 | 2 | Not known |
| DS4 | DS | 52 | M | 24 | 6 | bronchopneumonia |
| DS5 | DS | 64 | M | 31 | 6 | Not known |
| DS6 | DS | 66 | F | 26 | 5 | Not known |
| DS7 | DS | 67 | M | 8 | 5/6 | AD, CVD |
| DS8 | DS | 52 | F | - | 6 | AD, Lewy body dementia |
| DS9 | DS | 52 | M | - | 5 | TBI |
| DS10 | DS | 76 | F | 18 | 6 | Dementia |
| **Alzheimer’s disease** |  |  |  |  |  |  |
| AD1 | AD | 86 | F | 86 | 6 | Urinary tract infection, advanced dementia |
| AD2 | AD | 88 | M | 81 | 6 | Urinary tract infection, Addison’s disease, poor immunity, vascular dementia, AD |
| AD3 | AD | 83 | M | 46 | 6 | Bowel ischemia, hypothyroid, hypertension, AD, atrial fibrillation, chronic kidney disease, vascular dementia |
| AD4 | AD | 88 | M | 22.3 | 5 | Pneumonia, aortic stenosis, mixed dementia, left cerebellar hemisphere hemorrhage |
| AD5 | AD | 70 | M | 71 | 6 | Pneumonia, AD |
| AD6 | AD | 79 | F | 45.3 | 6 | Cerebrovascular accident, dementia |
| AD7 | AD | 78 | M | 62 | 6 | AD |
| AD8 | AD | 89 | M | 44 | 5 | AD |
| AD9 | AD | 78 | M | 24 | 4 | AD |
| AD10 | AD | 95 | M | 61 | 1 | Dementia |
| **Controls** |  |  |  |  |  |  |
| C1 | Normal | 66 | M | 10.3 | 5 | Cerebrovascular disease, dementia |
| C2 | Normal | 45 | F | 43.3 | 0 | End stage renal failure, diabetic nephropathy |
| C3 | Normal | 54 | F | 10.3 | 0 | Metastatic myxoid liposacroma, bronchopneumonia |
| C4 | Normal | 52 | F | 30.3 | 1 | Bronchogenic cancer |
| C5 | Normal | 75 | F | 24 | 2 | Ovary cancer |
| C6 | Normal | 66 | F | 29.3 | 2 | Metastatic breast cancer |
| C7 | Normal | 83 | M | 45 | 0 | Not known |
| C8 | Normal | 68 | M | 48 | 0 | Not known |
| C9 | Normal | 60 | F | 60 | 0 | Not known |
| C10 | Normal | 66 | M | 74 | 0 | Not known |

AD, Alzheimer’s disease; CVD, cerebrovascular disease; DS, Down’s syndrome; F, female; h, hour; M, male; TBI, traumatic brain injury

**Supplementary Table 2.** List of the primary antibodies used for immunohistochemistry

| Antibody | Species | Dilution | Supplier/cat. Number |
| --- | --- | --- | --- |
| Anti-β 42 (6E10) | Mouse (monoclonal) | 1: 1000 for IHC | Covance Cat Number  (SIG 39320) |
| Anti-β 42 | Rabbit (monoclonal) | 1: 1000 for IHC | Abcam (201060) mOC64 |
| Anti-Aβ40 | Rabbit (polyclonal) | 1: 500 for IHC | Thermo Fisher Scientific  (44348A) |
| Anti-APOE | Mouse (monoclonal) | 1: 1000 for IHC | Abcam (ab1907) |
| Anti-APOE | Rabbit (polyclonal) | 1: 200 for IHC | Abcam (ab85311) |
| Anti-TREM2 | mouse (monoclonal) | 1: 1000 for IHC | Abcam (Ab201621) |
| Anti-TREM2 | Rabbit (monoclonal) | 1: 1000 for IHC | Abcam (Ab209814) |
| Anti-TREM2 | Rabbit (polyclonal) | 1: 200 for IHC | Sigma-Aldrich (HPA010917) |
| Anti-total-Tau (HT7) | Mouse (monoclonal) | 1: 500 for IHC | Thermo Fisher Scientific  (MN1000) |
| Anti-Phospho-Tau (AT8, Ser 202, Thr205) | Mouse (monoclonal) | 1: 1000 for IHC | Invitrogen by Thermo Fisher Scientific (MN1020) |
| Anti-Myelin basic protein (MBP) | Rabbit (monoclonal) | 1:1000 for IHC | Abcam (Ab155995) |
| Anti-Iba1 | Rabbit (polyclonal) | 1:500 for IHC | Wako (019-19741) |

IHC, immunohistochemistry
